# Founder Management and Innovation: An Empirical Analysis Based on the Theory of Planned Behavior and Fuzzy-Set Qualitative Comparative Analysis

**DOI:** 10.3389/fpsyg.2022.827448

**Published:** 2022-02-14

**Authors:** Chun-Ai Ma, Rong Xiao, Heng-Yu Chang, Guang-Rui Song

**Affiliations:** ^1^School of Economics and Management, China University of Petroleum, Beijing, China; ^2^School of Business, Chang Gung University, Taoyuan City, Taiwan

**Keywords:** founder management, innovation, theory of planned behavior (TPB), fuzzy-set qualitative comparative analysis (fsQCA), investment

## Abstract

Based on the expanded theory of planned behavior, this study first explores the configuration relationship between founder management and innovation by using the fuzzy-set qualitative comparative analysis (fsQCA). Based on the theory of planned behavior, this study divides the behavior intention of founders into three categories: Attitude, subjective norm, and perceived behavior control. Using fsQCA, we found that there are two ways to achieve high innovation input of enterprises. In combination with the two ways, the factors such as male and highly educated founder, and large firm size can effectively increase the innovation input of firms, which is consistent with the three aspects of the behavioral intention of the theory of planned behavior, and it proves that the theory of planned behavior can effectively explain the configuration relation between the founder and firm innovation. In addition, this study finds that the innovation output is different from the innovation input, is dependent on the innovation ability of the firm itself, and is less influenced by the external environment.

## Introduction

The innovation literature suggests that unprecedented health emergencies, such as coronavirus disease 2019 (COVID-19), have the effect of stimulating firms to innovate (Ferrigno and Cucino, 2021). As a continuous power and inexhaustible source of organizational development, innovation is of great significance to the long-term development of the firm. However, due to its high risk and uncertainty regarding the outcome, innovation requires managers to have a sense of adventure and long-term vision. Managers tend to be shortsighted, reducing the level of innovation to avoid risk and improve short-term performance (Cho and Kim, 2017). Founders are often considered to be different from ordinary managers (Block, 2012). For example, Apple was criticized after the death of its founder, Steve Jobs, for failing to innovate in substance. Hence, it is of great significance for the development of firms to study the innovation behavior of founders.

In recent years, several research studies have examined the founder management on organizational innovation. For example, some scholars suggested that the founder-CEO-managed firm has a positive effect on innovation (Block, 2012; Lee et al., 2020). However, Xu et al. (2019) found that non-founder management of firm innovation input significantly increased, and research conclusions have been inconsistent. Independent variables and dependent variables are not uniformly symmetric, potentially explaining some of the inconsistencies in the findings (Rihoux and Ragin, 2009). Previous studies on founder management and innovation have relied on traditional regression methods, focusing on the net effect of variables (Lee et al., 2020). But the influence of the founder management on the innovation is abundant and complex, which also depends on the interaction of founders with the external environment (Schein, 1995).

Despite these, we noted that extant research has been limited to the study of management attributes of each founder in isolation and has neglected to analyze the relationships and interactions between attributes themselves. Understanding this issue is important for two reasons. First, the importance of the interactions among attributes of founders has been recognized as crucial for the survival prospects of start-ups (Nelson, 2003; Fahlenbrach, 2009). In fact, many authors advocate that the exploration of interactions among management attributes of founders contributes to creating a unique asset that reinforces the competitive advantage of the start-up (Certo et al., 2001; Nelson, 2003). Second, the inherent information asymmetry and uncertainty surrounding the innovation of firms makes it even more likely to need that an analysis of synergistic interactions among the management attributes of founders. For instance, it might show whether the synergistic interactions of the management attributes of founders amplify the influence of executives on company innovation (Liu et al., 2021).

To address these research gaps, our study focuses on how the combination of characteristics of founder management firms influences their innovation. One of the most prominent methods is the fuzzy-set qualitative comparative analysis (fsQCA) that is used with increasing frequency particularly in entrepreneurship and innovation-related studies (e.g., Cho and Kim, 2017; Kraus et al., 2018). When causality in the research phenomenon is multiple, an outcome has more than one cause, and these causes work together to produce the outcome, fsQCA represents an appropriate method (Kraus et al., 2018). Based on the developed theory of planned behavior, we found founder management and firm innovation are synergistic interactions. Hence, we used the fsQCA method to provide an alternative but complementary explanation to related research.

In our analyses, based on the expanded theory of planned behavior, this study first explores the configuration relationship between founder management and innovation by using fsQCA. Based on the theory of planned behavior, this study divides the behavior intention of founders into three categories: Attitude, subjective norm, and perceived behavior control. Using fsQCA, we found that there is no single exclusive causal path leading to the outcome and there are two ways to achieve high innovation input of firms. In combination with the two ways, the conditions such as male and highly educated founders, and large firm size can effectively increase the innovation input of firms, which is consistent with the three aspects of the behavioral intention of the theory of planned behavior, and it proves that the theory of planned behavior can effectively explain the configuration relation between the founder and firm innovation. In addition, this study finds that the innovation output is different from the innovation input, is dependent on the innovation ability of the firm itself, and is less influenced by the external environment.

Our study makes several important contributions to the literature on founder CEOs, innovation, and corporate governance. First, we contributed to the entrepreneurship literature, in general, and to the founder CEO literature, in particular (Fahlenbrach, 2009; Lee et al., 2020), by linking founder CEOs with innovation.

Second, we added to the innovation literature by using a new method to explain the relationship between founder management and innovation. QCA is a more suitable methodology to capture the impact of the interactions among variables on an outcome (Ragin, 1987), which distinguishes it from traditional quantitative and qualitative methods. In our study, we argued that fsQCA is particularly adequate for examining the relationship between innovation and entrepreneurship.

Third, we developed the theory of planned behavior and find it is suited for explaining entrepreneurship and innovation-related studies. The theory of planned behavior explains individual behavior from the perspective of psychology (Jindal and McAlister, 2015). We developed the theory of planned behavior by considering the complex external environment, intention of founders, and so on. This insight contributed to the theory of planned behavior highlighting how the synergistic interaction of founder management influences innovation of start-ups.

## Theory Development

Research has suggested that personal characteristics of CEOs and psychological attributes play important roles in determining the pursuit of innovation of a firm (Highfield and Smiley, 1987; Cho and Kim, 2017). The theory of planned behavior studies individual behavior from the perspective of psychology, and a central factor in the theory is the intention of an individual to perform a given behavior (Adams et al., 2009). The intention of an individual is assumed to capture the motivational factors that influence a behavior. Generally, the stronger the intention to engage in a behavior, the more likely should be its performance (Barker and Mueller, 2002; Bandiera et al., 2020). The theory of planned behavior has specified three determinants to explain how intentions engage in a specific behavior (Lee and Bae, 2020). The first determinant, attitude, describes the overall evaluation of the behavior of an individual. The second determinant, subjective norms, reflects the perceived social pressure regarding the performance of the behavior. Finally, the third predictor, perceived behavioral control, refers to the degree to which performing the behavior is perceived as easy or difficult. According to the theory of planned behavior, having a positive innovation-related attitude, strong innovation-related subjective norms, and high innovation-related perceived behavioral control increase the intention of an individual to engage in innovation behaviors (Jindal and McAlister, 2015; Straatmann et al., 2018).

**FIGURE 1:**
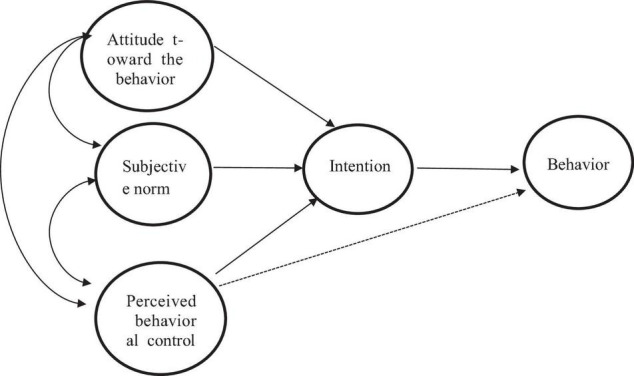
Theory of planned behavior.

What is more, it should be clear, however, that a behavioral intention can find expression in behavior only if the behavior in question is under volitional control (Zahra et al., 2008), i.e., the performance of most depends at least to some degree on such non-motivational factors as availability of requisite opportunities and resources (e.g., time, money, skills, and cooperation of others). Collectively, these factors represent actual control of people over the behavior. To the extent that a person has the required opportunities and resources, and intends to perform the behavior, he or she should succeed in doing so, and Figure 1 depicts the theory in the form of a structural diagram.

This study attempts to examine the complex relationship between founder management and firm innovation. As we studied how the individual behavior intention is transmitted to the specific firm innovation decision-making, the theory of planned behavior is quite appropriate (Jensen and Meckling, 1976; Haveman and Khaire, 2004; Kaplan et al., 2012; Ganter and Hecker, 2014). However, the theory of planned behavior needs to be expanded, and based on the research (Ma, 2009), the behavior intention of the individual founder can be transformed to the organizational decision-making of the firm, which depends on the dominant status of the founder in the firm, and this study extends Path 1, i.e., the behavior intention of the founder is adjusted by the status of the founder in the firm, which affects the organizational behavior of the firm.

**FIGURE 2:**
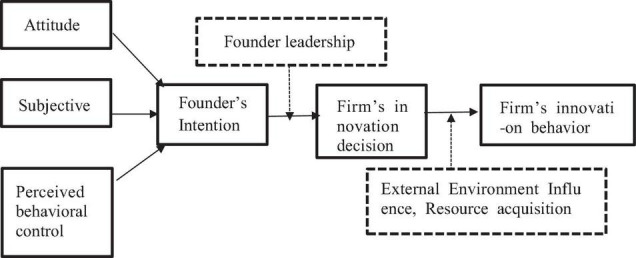
The extension of the theory of planned behavior.

In addition, whether the individual behavioral intention of the founder can ultimately influence the innovation-decision of the firm is also constrained by the complex external environment. To better understand the relationship of the role of founder to the firm, we require research and develop theory at three levels of analysis: individual, organizational, and environmental (Chandler and Hanks, 1994). According to the resource dependence theory, the survival of the organization needs to absorb resources from the surrounding environment, and it needs to interact with its environment to achieve its goal. Therefore, drawing on the research (Ma, 2009), this study extends Path 2. The influence of founders on innovation is also influenced by their ability to access resources, depending on the external environment. Figure 2 depicts the extension of the theory of planned behavior.

According to the theory of planned behavior, this study examines the relationship between founder management and firm innovation from three perspectives. From the perspective of attitude, relevant studies (Lan et al., 2020) show that risk tolerance is an important subjective factor that influences innovation decision-making of founders. Meanwhile, scholars found more educated executives have greater cognitive complexity (Wally and Baum, 1994; Hitt et al., 1997), which could influence the overall evaluation of the individual of the innovation behavior and the tendency toward accepting innovation. Therefore, this study selects the risk tolerance of the founders and education degree to measure their attitude to innovation.

From the perspective of subjective norms, subjective norms influence intentions because of their compliance function (Kelman, 1974), motivating the individual to act in a manner that will gain approval from those important to the individual (Eagly and Chaiken, 1993). This study measures subjective norms by whether the CEO is also founder of firm and the gender of the founder. If the CEO is also the founder, his or her decisions are subjected to pressure from stakeholders inside or outside the company. In addition, CEO attributes matter for innovation, and their compliance function is amplified if the CEO is also a founder (Lee et al., 2020). Some studies have also pointed out (Cooper, 2012) that the external pressure and moral constraints, which male and female managers bear are different, will directly affect their decision-making behavior. These variables could better measure the perceived social pressure regarding the performance of the founder innovation behavior.

From the perspective of perceived behavioral control, we used the scale of firm and political relevance to measure. Perceived behavioral control refers to the degree to which performing the behavior is perceived as easy or difficult. The founder has served or is serving as a deputy in government which can send a positive signal to the outside world, helping companies to reduce financing constraints in innovative financing (Kim and Lu, 2011). At the same time, based on the resource-advantage view, large firms are assumed to be more resourceful and proactive (Russo and Fouts, 1997; Aragon-Correa et al., 2008), and small firms lack skills, capabilities, and financial and human resources (Biondi et al., 2000; Bowen, 2000). This theoretical view implies that the size of firms also represents the ability of firms to access resources. Moreover, these two indicators can also reflect the external environment and resource constraints faced by firms, which will influence the innovation behavior perceived as easy or difficult.

## Research Method

### Data Analysis Methods

To answer our research question, the QCA method has been used. More specifically, four reasons led us to use this method. First, fsQCA bridges quantitative and qualitative approaches and allows us to analyze causal relationships between configurations (Ragin, 2008). As entrepreneurial innovation and founder management are asymmetric, management attributes of the founder of synergistic interactions may amplify the influence of executive on company innovation (Liu et al., 2021), and fsQCA can supplement regression analysis. Second, fsQCA is recently applied increasingly in entrepreneurship and innovation-related studies (e.g., Mas-Verdú et al., 2015; Kraus et al., 2018). Furthermore, fsQCA can account for equifinality, i.e., a situation in which an outcome may follow from different combinations of causal conditions, i.e., from different causal “recipes” (Ragin, 2008). Last, the aim of this method is not to reveal patterns that support the existence of the causal relationship but rather to identify whether some configurations are associated with an outcome of interest (Wasserman, 2003). Notably, fsQCA assumes cases as combinations of different values for the outcome of interest and the causal conditions. Accordingly, it was suitable to research the combination of different founder management “routes” that lead to firm innovation.

### Sample

Founder management is more typical in private companies, so in the part of researching innovation input, the sample is derived from the 2019 IPO data of private company in the China A-share market. Since 2017 is the most recent year for which data are available to measure the patent output of firms in the CSMAR database, the measurement period of innovation output is 2017. Furthermore, we excluded firms with missing financial data, firms in the financial industry, ST and PT firms, and firms whose asset-liability ratio is greater than one, the final innovation input research got 328 samples of private listed companies, and innovation output research got 635 samples of private listed companies for study.

The data in this study are from the CSMAR database and WIND database. The data were analyzed using FSQCA3.0 software.

### Outcome

#### Innovation

We followed prior literature and measured R&D productivity by the effect of R&D spending (Moshirian et al., 2020). Specifically, we used the logarithm of R&D cost plus one (Ln_R&D) to measure innovation input.

This study selects the patent output of firms as the main index to measure the innovation output. There are three kinds of patents in China: invention patent, utility model patent, and design patent. Since design patents do not involve innovation, this study uses the research by Tian and Meng (2018) for reference and chooses the sum of invention patents and utility model patents (Patents 1 and 2) to measure firm innovation output. The patent data in this study comes from the patent database of listed companies and subsidiaries of CSMAR, which can measure the innovation of firms more comprehensively.

### Conditions

#### Founder Management (fc)

Drawing on the existing study (Xia et al., 2012), the founder management data in this study are collected manually. According to the description of “issuer status” in the prospectus of each sample company, we found out which person or group of people started the business in the first place. For a company founded by more than one person, the founder who plays the most important role (holding the most shares before the issue, or serving as chairman or general manager during the initial establishment of the company, etc.) is considered as the founder.

After identifying the founders, we obtained the names of the chairman and general manager (also known as president, CEO, etc.) of the company at the time of its listing from the CSMAR corporate governance database and checked with the name of the founder to determine if the CEO is also the founder of the company. “Founder management” is a virtual variable. If the CEO is also a founder, the value of founder management is 1; otherwise, it is 0.

We measured the gender of the founder (Gender) by creating a dummy variable coded as 1 if the founder is a man and 0 for a woman. Drawing on the research of Wang and Wang (2019), founder education was divided into a five-point scale: 0, junior high school and below; 1, senior high school; 2, junior college; 3, undergraduate degree; 4, master degree; and 5, Ph.D. degree. We also measured founder political affiliation (Gl) by creating a dummy variable coded as 1 if the founder has served or is serving as a deputy in government; otherwise, a variable coded as 0. Firm size is measured as natural logarithms of total assets. Based on the studies by Coles et al. (2006) and Lan et al. (2020), and upper echelons theory, this study uses short-term solvency (net working capital/total debt) to measure founder risk tolerance. Table 1 presents variable design.

**TABLE 1:** Variable design.

Variable classification	Variable	Variable code	Definition
Innovation behavior	Innovation input	Ln_ R&D	Logarithm of R&D cost plus one
	Innovation output	Patent1&2	The sum of invention patents and utility model patents
Attitude	Risk tolerance	Fx	Net working capital/total debt
	Founder education	xl	Junior high school and below, set at 0; senior high school, set at 1; junior college, set at 2; undergraduate, set at 3; master, set at 4; and doctor, set at 5.
Subjective	Founder management	fc	If the CEO is founder, the value of founder management is 1, Otherwise 0.
	Founder gender	Gender	The founders are set to 1 for men and 0 for women.
Perceived behavioral control	Firm size	Size	Ln (Total assets)
	Founder political affiliation	gl	If the founder has served or is serving as a deputy in government, set it at 1; Otherwise, set it at 0.

### Calibration

In fsQCA, the outcome to be explained and the different causal conditions are assumed to range from no membership to full membership in a given set condition. Full membership is denoted by a value of 1 and no membership with a value of 0. Intermediate values, which denote partial membership in a set condition, are given values between 1 and 0. Membership scores greater than 0.5 indicate that a case is “more in than out” in the set condition, scores close to 1 indicate that a case is “mostly in” a set condition, scores close to 0 indicate that a case is “mostly out,” and so on. It requires substantiation of the method of “calibration,” i.e., the transformation of original data to a scale over the interval (0, 1) (Ragin, 2008).

In this study, the upper quartile (75%), median (25%), and lower quartile (25%) of the descriptive statistics of the case samples are set up for 6 conditional variables and 2 outcome variables to calibrate. Tables 2, 3 present calibration anchors and descriptive statistics for each variable.

**TABLE 2:** Innovation input calibration and descriptive statistics.

	Calibration			Descriptive statistics			
	Completely in	Point of maximum ambiguity	Completely out	Mean	*SD*	Minimum	Maximum
Gender				0.633	0.250	0	1
fc				0.479	0.500	0	1
gl				0.195	0.396	0	1
Size	20.721	21.119	21.611	21.234	0.746	19.665	25.342
xl	2	3	4	3.238	1.178	0	5
fx	0.453	1.085	2.509	1.941	2.339	–0.326	16.368
Ln_R&D	17.117	17.670	18.214	17.713	0.943	13.442	21.819

**TABLE 3:** Innovation output calibration and descriptive statistics.

	Calibration			Descriptive statistics			
	Completely in	Point of maximum ambiguity	Completely out	Mean	*SD*	Minimum	Maximum
Gender				0.632	0.251	0	1
fc				0.510	0.500	0	1
gl				0.249	0.432	0	1
Size	20.543	20.960	21.555	21.094	0.748	19.647	24.616
xl	2	3	4	2.976	1.230	0	5
fx	0.775	1.630	3.226	2.432	2.613	–0.749	18.920
Patent1&2	1.609	2.485	3.238	2.365	1.337	0	7.517

### Fuzzy-Set Qualitative Comparative Analysis

The fsQCA involves three steps (Ragin, 2006). The first step consists of constructing a truth table, which reports all logically possible combinations of conditions and the outcomes associated with each configuration. Each row shows one of the logically possible combinations of conditions (Sui and Baum, 2014). The second step reduces the number of rows in the truth table considering two conditions, namely, a frequency threshold and a consistency threshold (Sui and Baum, 2014). Following Wasserman (2003), we applied a frequency threshold of 1 and a coherence threshold of 0.8, respectively. The third step uses an algorithm to simplify the truth table. In our study, we used the Quine McCluskey algorithm (used in the fsQCA 3.0 software package) to obtain a more parsimonious response.

## Empirical Results

### Analysis of the Necessity Conditions

The necessity analyses evaluated whether a condition must be presented for an outcome to occur. Consistency is the degree to which a given solution is a subset of the outcome, and coverage is the degree to which the outcome can be interpreted by a given solution, like *R*^2^ in regression analysis. The coverage is divided into raw coverage and unique coverage. We further reported raw and unique coverage measures. Raw coverage of a respective solution term is the coverage if only the respective solution term is assumed to be present. Unique coverage expresses the contribution of a solution term beyond what is explained already by other terms. This section presents the results of necessity analyses for the conditions gender, namely, gl, fc, fsxl, fsfx, and fsize. For the necessity analyses of conditions for innovation outcome Ln_ R&D and Patents 1 and 2, refer to Table 4.

**TABLE 4:** Necessity analyses for innovation input and output.

Condition	High innovation input		High innovation output	
	Cons	Cov	Cons	Cov
Gender	0.951	0.515	0.930	0.498
∼Gender	0.049	0.369	0.070	0.519
gl	0.174	0.451	0.251	0.503
∼gl	0.826	0.518	0.749	0.498
fc	0.498	0.525	0.542	0.531
∼fc	0.502	0.486	0.457	0.466
fsxl	0.457	0.615	0.513	0.559
∼fsxl	0.648	0.523	0.597	0.550
fsfx	0.510	0.503	0.492	0.480
∼fsfx	0.588	0.607	0.596	0.610
fsize	0.736	0.741	0.651	0.643
∼fsize	0.362	0.367	0.442	0.446

The standard threshold of consistency value is 0.90 (Ragin, 2008). As Table 4 shows, gender is a necessary condition and, in China, this may be due to men accounting for the clear majority parts in private firms. Furthermore, we considered the influence of configuration.

### Analysis of the Sufficiency Conditions

The analysis of sufficiency identifies all the conditions that are sufficient for the result to occur. Table 5 provides the main results of sufficiency analyses, which consider sets of conditions. These conditions lead to the outcome.

**TABLE 5:** Sufficiency analyses results for high innovation input and output.

High innovation input			High innovation output
Conditions	Solution 1	Solution 2	Solution 1
Gender	•	🌑	🌑
fc		🌑	🌑
gl	•	•	•
Size	🌑	🌑	•
xl	🌑	🌑	🌑
fx	⊗	🌑	🌑
Consistency	0.833514	0.836181	0.834215
Raw coverage	0.278207	0.150761	0.029842
Unique coverage	0.196484	0.069039	0.029842
Solution consistency	0.822107		0.834215
Solution coverage	0.347246		0.029842

*🌑 Indicates the existence of a core condition, ⊗ indicates a lack of core conditions, • indicates the existence of a peripheral condition, while blank cells represent “don’t care” conditions.*

#### Sufficiency Analyses Results for High Innovation Input

According to previous literature (Schneider and Wagemann, 2012), we adopted a coherence threshold of 0.80 for judging a correspondence with necessity/sufficiency hypotheses as sufficient.

The fsQCA can produce three solutions: complex solution (not including logical remainder), intermediate solution (only including logical remainder), and parsimonious solution (including all logical remainders). According to the study by Ragin (2008), the intermediate solution is indicated as the most suitable since it achieves a balance between the complex solution and the parsimonious solution in terms of complexity. The intermediate solution is, therefore, a subset of the other possible solutions, namely the complex solution and the parsimonious solution (Wasserman, 2003). Therefore, in this study, the condition that exists in both the parsimonious solution and the intermediate solution is regarded as the core condition, and the condition that exists only in the intermediate solution is regarded as the peripheral condition.

Table 5 presents the results of the fsQCA. The solution table exhibits two solutions achieving high innovation input. As mentioned in Table 5, the overall consistency is 0.82, higher than the consistency standard of 0.8, and the consistency of each configuration is also higher than 0.8.

Solution 1 combines the presence of a highly educated founder and large firm size with the absence of risk tolerance of the founder, supplemented by the strong political connections of the founder and male gender of the founder. Core conditions are the presence of a highly educated founder, large firm size combined with the absence of risk tolerance. Thus, this configuration shows that when the risk tolerance of the founder is insufficient, the cognitive condition and perceived behavioral control condition are decisive in leading to high innovation input.

Solution 2 shows that the combination of large-sized firms with male, highly educated, highly risk-tolerant founder as CEO, supplemented by the fact that political connections is more conducive to the innovation input of the firm. Compared with solution 1, solution 2 increases the risk tolerance of the founder, the founder as CEO, and gender as core conditions.

Taken together, solutions 1 and 2 suggest that the factors such as the founder is highly educated and large firm size together can effectively enhance the innovation input of the firm. The founder is aware of the importance of innovation input to the long-term development of the firm. At the same time, since the founder usually owns a high share of the firm, this role has both motivation and power to increase the innovation input of the firm. Based on the theory of planned behavior, these conditions are subordinate to three factors: Attitude, subjective norm, and perceived behavior control, which further proves that the theory of planned behavior can effectively explain the innovative investment behavior of founders. A lot of psychological and behavioral economics research shows that women are more conservative when it comes to risk-taking, and innovation investment is a high risk and uncertain investment, male founders are more confident when it comes to making risky decisions. Moreover, highly educated founders obtain more innovation-relevant knowledge, which will reduce the information asymmetry problem in the process of investment in organizational innovation (Block, 2012). They have a long-term vision for the development of the organization, which will pay more attention to innovation investments. While the political affiliation of the founders and the large size of the firm could help organizations gain resources much easier, the firms may face relatively few obstacles when they carry out innovation financing. Besides, Manso (2011) found that risk tolerance of entrepreneurs is an important factor that affects the innovation of organizations. In addition, the fact that CEO is also the founder can increase the innovation input of the organization, which means the founder can directly manage the firm, because of his or her unique status, better supervise and manage the innovation investment of the firm, and reduce the agency cost; it also shows that the impact of the founder on the innovation investment of the company depends on his or her position in the company.

#### Sufficiency Analyses Results for High Innovation Output

Looking at the group of high innovation output, the fsQCA produces a group of solutions and the total consistency is 0.83, which is higher than the consistency standard of 0.8. As mentioned in Table 5, the combination of male, highly educated, highly risk-tolerant founder as CEO in the company leads to high innovation output of the firm, supplemented by the political connections of the founder and the large size of the firm. Different from innovation input, it is noted that in this group of innovation output, the attitude and the subjective norm perspective in the theory of planned behavior play a leading role, and the impact of the external environment and resource acquisition capacity may not be so important, which indicates that the innovation output depends more on the innovation capacity of firms themselves.

### Robustness

To ensure the robustness of the results, we raised the consistency to 0.85 and then analyzed again, as shown in Table 6. We also made a robustness test on the adjustment of the frequency threshold and reported the results after the adjustment of the consistency.

**TABLE 6:** Robustness.

	High innovation input		High innovation output

Conditions	Solution 1	Conditions	Solution 1
Gender	🌑	Gender	🌑
fc	🌑	fc	🌑
gl	•	gl	•
Size	🌑	Size	•
xl	🌑	xl	🌑
fx	🌑	fx	🌑
Consistency	0.891233	Consistency	0.834215
Raw coverage	0.0816623	Raw coverage	0.029842
Unique coverage	0.0816623	Unique coverage	0.029842
Solution consistency	0.891233	Solution consistency	0.834215
Solution coverage	0.0816623	Solution coverage	0.029842

*🌑 Indicates the existence of a core condition, • indicates the existence of a peripheral condition, while blank cells represent “don’t care” conditions.*

As can be seen from Table 6, the solution is basically the same after raising the consistency, which proves that the conclusion of this study is reliable and robust.

## Conclusion: Contributions, Limitations, and Future Research

How to improve the innovation input and innovation output of firms is the focus of this innovation research. In this study, the theory of planned behavior is extended, and the method of fsQCA is used for the first time to discuss the configuration effect of organizational innovation from the view of founder management. This study finds that three perspectives of the theory of planned behavior, namely, attitude, subjective norms, and perceived behavior control, can effectively explain the relationship between founder management and organizational innovation. Two different solutions can lead to high innovation input. First, the configuration takes the presence of highly educated founder, large firm size and the absence of risk tolerance as core conditions leads to high innovation input. Second, the combination of large firm size with having a male, highly educated, highly risk-tolerant founder, who coupled with being CEO and having strong political connections, are more conducive to increasing the innovation investment of the firm. Combining the theory of planned behavior, these two paths both contain three factors that influence the behavior intention of the founder, and the second path shows that the founder takes the role of CEO, which shows that the founder plays a leading role in the decision-making of the organization, and the behavior intention of the founder can effectively rise to the innovation decision-making of the firm and, finally, form the innovation input behavior of the firm under the influence of the two external environments of political connection and firm size. We determined that the male, highly educated, highly risk-tolerant founder as the CEO are core conditions for innovation output. This configuration shows that the innovation output is different from the innovation input and more dependent on the transformation of internal resources, but less affected by the external environment and resource acquisition capability.

This study provides an empirically validated framework to explore founder management and innovation. First, based on the expanded theory of planned behavior, this study explores the complex relationship between founder management and organizational innovation behavior. Earlier, most of the literature on the founder was simply set as a virtual variable (Kim and Lu, 2011; Markin et al., 2021), without considering the personal characteristics of the founder and how the personal intention of the founder rose to corporate behavior. This study expands the theory of planned behavior, which is mainly used in the fields of psychology and sociology, opens the black box between the founder and the innovation behavior of firms, and describes the path that the behavior intention of the founder forms the innovation decision-making behavior of the firm.

Second, this study contributed to innovation and entrepreneurship studies (Fang et al., 2014; Falato et al., 2015; Cain and McKeon, 2016; Del Sarto et al., 2019; Ferrigno et al., 2021). The previous literature neglected the influence of the interaction between the personal characteristics of the founder and the external environment as well as the “chemical reaction” of their combination on the innovation behavior of a firm (Dell’Era et al., 2020). This study examines the relationship between founder and innovation from the perspective of configuration by using the method of fsQCA, and it provides a new way of thinking for the research of firm innovation field.

Third, we offered a methodological contribution. This study finds that the planned behavior theory and the fsQCA approach are highly matched in exploring the relationship between founders and firm innovation, and it is further proved that the combination of the theory in the field of psychology and the method in the field of management can effectively explain the problems of management practice and has positive significance to the research in the field of management.

The founders are vital to the long-term growth of the company. Most innovative companies we know are also under the control of their founders (Google, Facebook, etc.). But the role of founders in business innovation is not just through controlling the business. This study demonstrates that expanding the size of the business, increasing the education of the founders, and strengthening the political connections of the founders, in order to improve the risk tolerance of founders, are helpful to increase firm innovation input, while the founder with high educational background, high-risk tolerance, and to be CEO are effective ways to improve the innovation output.

The study also offers implications for both policy and practice. First, founder managers should constantly improve themselves to achieve professional governance. For firm innovation, managers need to have an independent judgment of technological development. The founder improves own knowledge quality, which is helpful for the firm to formulate suitable innovative decision-making.

Second, the founder should build a social resource network to reduce the dependence on the external environment. The founder strengthens the communication and cooperation with the government, is advantageous in grasping the market in time, and obtains more innovation financing, then promotes the firm innovation high-quality development.

Moreover, several limitations must be taken into account about this study. First, due to the limitation of the sample size of fsQCA, this study used only the data of the past 1 year, lacked the study of the dynamic change, and did not carry out the cross-time comparison, and the data were collected only from Chinese database and the manual collection; in the future, we can use the form of questionnaire from other countries for further analysis. In addition, firm innovation is an extremely complex firm behavior, which is affected by many factors. This study analyzes only some configurations with the theory of planned behavior, and future studies could investigate these configurations in different contexts.

## Data Availability Statement

The datasets presented in this study can be found in online repositories. The names of the repository/repositories and accession number(s) can be found below: China Stock Market and Accounting Research Database, https://www.gtarsc.com/.

## Author Contributions

C-AM: conceptualization, methodology, software, and writing—original draft preparation. RX: data curation, visualization, and investigation. H-YC: writing—reviewing and editing. G-RS: software and validation. All authors contributed to the article and approved the submitted version.

## Conflict of Interest

The authors declare that the research was conducted in the absence of any commercial or financial relationships that could be construed as a potential conflict of interest.

## Publisher’s Note

All claims expressed in this article are solely those of the authors and do not necessarily represent those of their affiliated organizations, or those of the publisher, the editors and the reviewers. Any product that may be evaluated in this article, or claim that may be made by its manufacturer, is not guaranteed or endorsed by the publisher.
